# Analysis of a gene co-expression network establishes robust association between Col5a2 and ischemic heart disease

**DOI:** 10.1186/1755-8794-6-13

**Published:** 2013-04-10

**Authors:** Francisco Azuaje, Lu Zhang, Céline Jeanty, Sarah-Lena Puhl, Sophie Rodius, Daniel R Wagner

**Affiliations:** 1 Department of Translational Cardiovascular Research, CRP-Santé, Luxembourg, Luxembourg; 2 Department of Internal Medicine III, Saarland University Hospital, Homburg, Germany; 3 Division of Cardiology, Centre Hospitalier, Luxembourg, Luxembourg; 4 Current Address: Department of Oncology, NorLux Neuro-Oncology Laboratory, CRP-Santé, Luxembourg, Luxembourg

**Keywords:** Systems-based approaches, Co-expression networks, Myocardial infarction, Collagen proteins, Col5a2

## Abstract

**Background:**

This study aims to expand knowledge of the complex process of myocardial infarction (MI) through the application of a systems-based approach.

**Methods:**

We generated a gene co-expression network from microarray data originating from a mouse model of MI. We characterized it on the basis of connectivity patterns and independent biological information. The potential clinical novelty and relevance of top predictions were assessed in the context of disease classification models. Models were validated using independent gene expression data from mouse and human samples.

**Results:**

The gene co-expression network consisted of 178 genes and 7298 associations. The network was dissected into statistically and biologically meaningful communities of highly interconnected and co-expressed genes. Among the most significant communities, one was distinctly associated with molecular events underlying heart repair after MI (P < 0.05). *Col5a2*, a gene previously not specifically linked to MI response but responsible for the classic type of Ehlers-Danlos syndrome, was found to have many and strong co-expression associations within this community (11 connections with ρ > 0.85). To validate the potential clinical application of this discovery, we tested its disease discriminatory capacity on independently generated MI datasets from mice and humans. High classification accuracy and concordance was achieved across these evaluations with areas under the receiving operating characteristic curve above 0.8.

**Conclusion:**

Network-based approaches can enable the discovery of clinically-interesting predictive insights that are accurate and robust. *Col5a2* shows predictive potential in MI, and in principle may represent a novel candidate marker for the identification and treatment of ischemic cardiovascular disease.

## Background

In the era of modern reperfusion therapies, acute myocardial infarction (MI) remains associated with substantial morbidity and mortality. MI is underpinned by complex, intertwined biological processes [1]. These processes operate in the context of large, intricate biological interaction networks. Despite over 60,000 reports on MI [2,3], there is still a pressing need to better define the disease biology of this condition based on integrative, systematic approaches. Indeed, systematic network-based approaches can bridge the gap between our knowledge of the functional roles of molecular entities, disease phenotypes and new clinical applications [4,5]. We and others have shown that such an approach may generate new targets and markers for MI, which may become clinically useful [6-9].

Crucial requirements should be met as necessary conditions to leverage the power of systems-based approaches: 1. Models should be capable not only to describe biological phenomena, but also to make predictions about phenomena; 2. The resulting predictive models should provide the basis for potentially novel, clinically-driven applications; and 3. model-based predictions should stand up to the test of independent validations.

At the center of our systems-based knowledge discovery strategy is the detection of functionally relevant network *communities*. A community, also often referred to as a *module*, is here defined as a group of genes that is both highly inter-connected and strongly co-expressed. We identified a weighted gene co-expression network in MI by estimating similar gene expression patterns across mice-derived samples. We implemented a new computational approach to network community detection, and searched for potentially clinically relevant communities, including those involving genes relatively uncharacterized in the context of MI. To demonstrate the predictive potential of our top prediction, we implemented computational models to distinguish MI from control samples using this gene’s expression data. After estimating the discriminatory capacity of this model on the network-generating dataset, we implemented an independent evaluation of the model on quantitative real-time PCR data. Additional independent validations of the classification model were successfully carried out on public microarray data.

In this investigation, we aimed to analyze a gene co-expression network of MI. This effort allowed us to: a. determine the potential predictive role of a relatively uncharacterized gene, *Col5a2*, and its associated transcriptional partners in MI; and b. demonstrate the disease discriminatory capacity and reproducibility of such network-derived insights.

## Methods

### Datasets

The co-expression network in MI was derived from a microarray dataset consisting of 36 MI and 23 control cardiac tissue samples published in Tarnavski *et al.*[10] (GEO accession code: GDS488). MI samples were obtained from mice that underwent ligation of the left coronary artery, and control samples originated from sham-operated mice. Details of experimental protocol are published in Tarnavski *et al.*[10]. Hereafter this dataset is referred to as the model *derivation* dataset.

We validated models on several independently generated datasets. First, we measured gene expression of *Col5a2* using qPCR data in MI and control samples (details are shown below). A second independent evaluation was performed on a (microarray) expression dataset (GDS2329) that consisted of 10 MI and 10 control samples from mice [11]. We also tested the disease discriminatory potential of *Col5A2* in human data from the Harvard’s CardioGenomics project (32 ischemic cardiomyopathy vs. 14 control samples) [12]. We note that the time between ligation and the acquisition of the samples varied across the different independent datasets. However, we emphasize that in our qPCR validation dataset the time between ligation and sample extraction was the same for all the mice.

### Animal model

To independently validate our findings, we first implemented a mouse model of MI as follows. MI was induced by ligation of the left anterior descending coronary artery (LAD). Control samples were obtained from sham-operated mice, which underwent the same surgery procedure as MI mice without occlusion of the LAD. Heart samples (left ventricular myocardium) were obtained 4 weeks after surgery in both groups: 15 MI and 6 control samples.

Mice were anesthetized with a 1:10 dilution (diluted with 0,9% NaCl) of a mixture of Ketaminhydrochlorid (100 mg/kg) and Xylazinhydrochlorid (10 mg/kg). Ten minutes after administration, movement of whiskers and reflexes was tested. Lack of reaction ensured a stable and deep sedation for about 40 minutes. Mice were euthanized by an intraperitoneal application of an undiluted mixture of Ketaminhydrochlorid (100 mg/kg) and Xylazinhydrochlorid (10 mg/kg). Details are available in Additional file 1.

The study was approved by the animal Ethics Committee of Saarland University, Germany, and animal handling was performed according to the European directive on Laboratory Animals (86/609/EEC) and the Guide for Care and Use of Laboratory Animals by the US National Institute of Health (NIH Publication No. 85–23, revised 1996).

### Quantitative real-time PCR experiments

Total RNA was extracted from frozen tissue samples with a Trizol (Invitrogen, Carlsbad, CA) isolation protocol. 1 μg of RNA were reverse transcribed into cDNA using the SuperScript II reverse transcriptase (RT). cDNAs were diluted 10-fold and 4 μL were mixed with 16 μL of SYBR®Green Master Mix (Biorad, Nazareth, Belgium) containing 300 nM of each primer (final volume 20 μL). After each run a melting curve analysis was analyzed, ranging from 55°C to 95°C in 20 min. A negative control without cDNA template was run in every assay and measures were performed in duplicates. Intron-flanking primers were designed with the Beacon Designer Pro 7.8 software (Premier Biosoft, Palo Alto,USA). Specificity was assessed using the NCBI BLAST tool [13]. Melting curves were analyzed and amplicons were observed on agarose gel to confirm the specificity of the reaction. HPLC-purified primers were obtained from TIB MOLBIOL (Berlin, Germany). Expression levels were calculated using the CFX manager 2.1 software (Biorad) via the delta-Cq method, incorporating the calculated amplification efficiency for each primers pair. GAPDH was used as reference gene. The mean raw Cq values were the inputs to the PCR data analysis. Details, including compliance with MIQE guidelines [14], are available in Additional files 2 and 3.

### Gene co-expression network: generation

**Figure 1:**
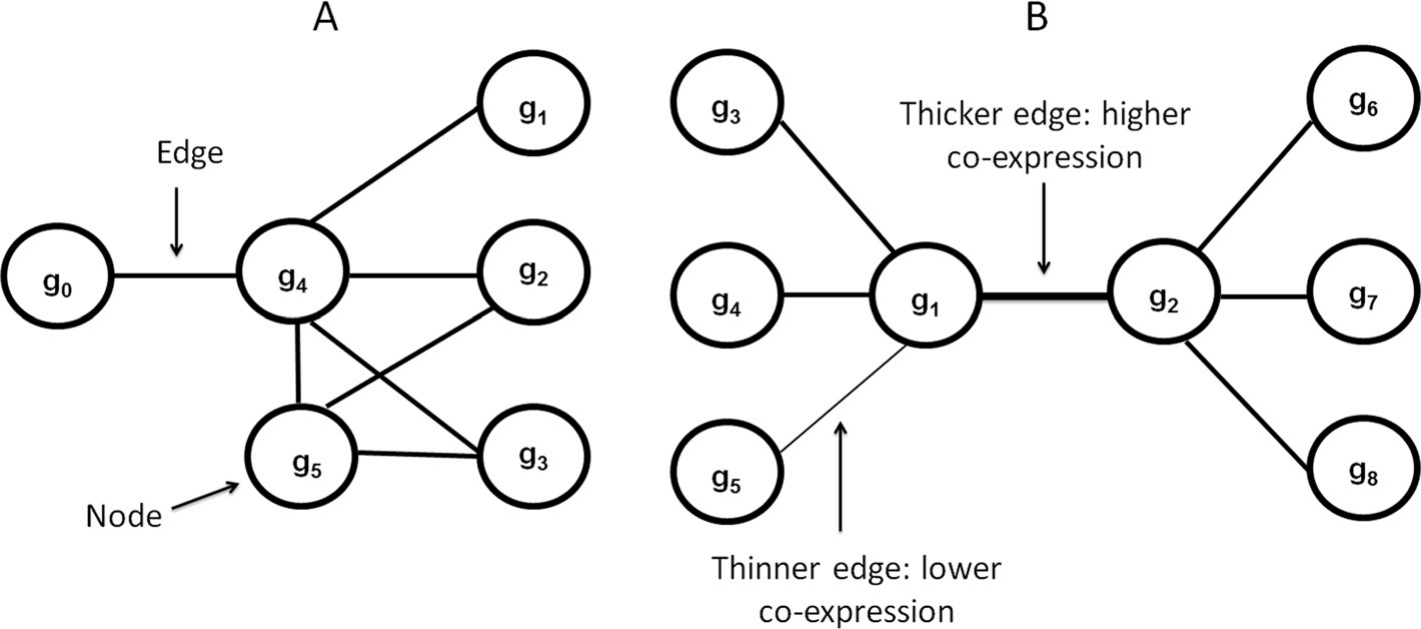
**Illustration of fundamental network analysis concepts. A** and **B** show hypothetical examples of candidate communities that can be detected by our approach. Nodes and edges represent genes and co-expression values respectively. The thickness of the edges can be used to graphically represent co-expression levels.

Flat expression patterns across samples in the derivation dataset were filtered out by excluding genes with standard deviations ≤ 0.1. Spearman co-expression coefficients, ρ, were calculated among all pairs of the remaining genes. All gene pairs with ρ ≥ 0.1 represented gene-gene associations in the network. A weighted gene co-expression network in which nodes and edges denote genes and co-expression values respectively was next generated. Figure 1 illustrates fundamental network concepts used in this article.

### Gene co-expression network: community detection

Candidate biologically meaningful communities were detected by applying A-CODE (association-centered community detection algorithm) (Additional file 1). This approach is based on the notion that strong communities are built around strong edges in the community. Moreover, candidate communities should also represent tightly interconnected webs of neighboring edges. Thus, A-CODE searches for strong, highly-interconnected communities around each edge in the network (examples in Figure 1). Candidate communities are characterized by their co-expression *compactness*, which is here based on the mean co-expression value observed in the candidate community. To reduce possible bias towards highly variable co-expression patterns, compactness is computed as the mean co-expression value divided by the standard deviation of the values found in a candidate community. The expected rate of false discoveries, *q*, for each observed compactness value is computed with a statistical test based on random permutations. Thus, strong candidate communities are those displaying high co-expression compactness with corresponding low *q* values. At each search step, A-CODE adds a new edge to the candidate community. Each new edge is derived from the direct neighborhood of the current candidate community. At each search step the neighboring edge with the highest co-expression value, ρ, is selected for inclusion. This process continues until either a minimum *q* (min_*q*) cannot be obtained or until a maximum number of edges in the candidate community has been reached. Experiments reported here are based on min_*q* = 1E-4, and minimum and maximum numbers of 5 and 20 edges respectively in each candidate community. The latter was suitable to assist expert visualization and interpretation. Also the min_*q* value selected is stringent enough to filter out communities for which more than 1 permutation experiment (out of 10000 implemented) reported compactness values equal or higher than that observed in the candidate community. At the end of this process, each network edge gives rise to a candidate community. Thus, unlike the conventional view of network clustering, a key feature of our approach is that it allows the identification of not only candidate communities formed around highly connected nodes, but also of candidate communities defined by highly connected, strong edges.

### Disease classification model

To demonstrate the disease discriminatory capacity of *Col5a2*, a classification model based on logistic regression was implemented (Ridge estimation value: 1E-08). Classification performance was assessed with areas under the receiving operating characteristic curve (AUCs). Using the derivation dataset, a classification model was built and its discriminatory capacity was first estimated with leave-one-out cross-validation. The resulting model was next tested on independent datasets using *Col5a2* as model input after standardization (mean value = 0, standard deviation = 1).

### Software tools

The derivation dataset was pre-processed with the Gepas tool [15]. Other datasets were pre-processed with the (R-platform) affy package [16]. The weighted co-expression network was generated with BioLayout [17] and visualized with Cytoscape [18]. We applied the DAVID tool to examine network candidate communities on the basis of their associations with functional annotations [19]. A-CODE was coded in Java (Additional file 1). Classification models were implemented in Weka [20]. Additional statistical analyses were completed with SigmaPlot [21]. Statistical significance of differential expression was estimated using Student’s t-test, and P values were adjusted for multiple testing using Benjamini & Hochberg test.

## Results

### A gene co-expression network in MI

**Figure 2:**
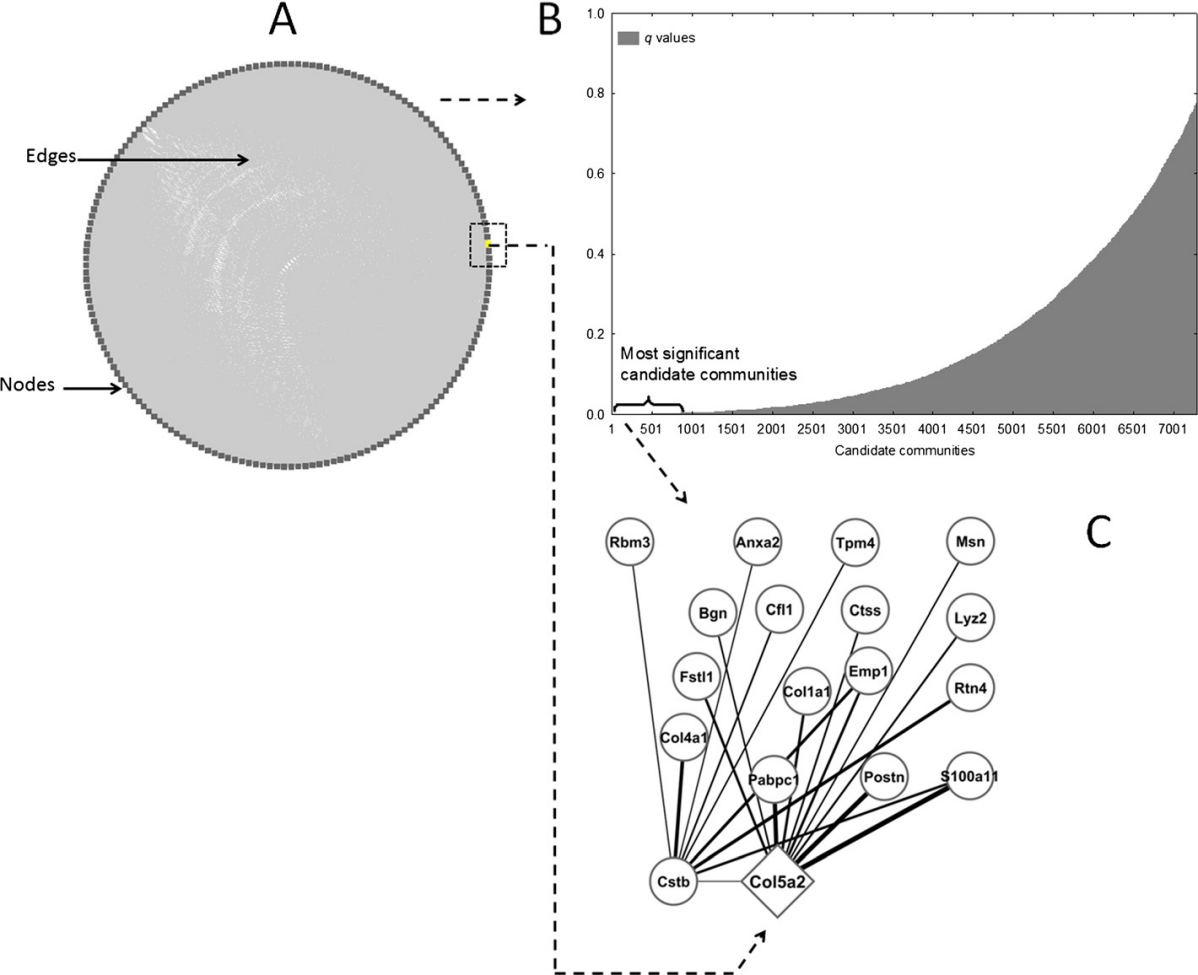
**Gene co-expression network in MI encodes clinically interesting knowledge. A**. Graphical view of the network. Nodes and edges represent genes and co-expression relationships respectively. Because genes are highly densely interconnected, edges are difficult to graphically discern, and here are shown as a grey area inside the (circle) network layout. **B**. Overview of the statistical landscape of network communities detected. The *q* values reflect the statistical relevance of the candidate communities. **C**. A highly interconnected and co-expressed community, in which *Col5a2* is shown as a potential relevant gene with predictive value. The thickness of the edges reflects the observed co-expression values.

We generated a co-expression network using the derivation dataset as outlined above. The resulting network consists of 178 nodes and 7298 edges highly interconnected as a single, large unit (Figure 2A, Additional file 4). As further illustrated by basic network topology parameters, genes are in relatively close proximity to each other and are tightly grouped (characteristic path length: 1.76, clustering coefficient: 0.92). This made analysis with standard network community detection techniques difficult. Our A-CODE algorithm revealed the complexity and potential relevance of the community structure of the network in more detail. As expected, the vast majority of candidate communities detected are statistically irrelevant (Figure 2B). Nevertheless, our approach detected hundreds of potentially interesting communities (*q* < 0.001) that exhibit highly transcriptionally compact patterns. Additional file 5 shows examples of top candidate communities.

### *Col5a2* has predictive value in cardiac repair after MI

**Figure 3:**
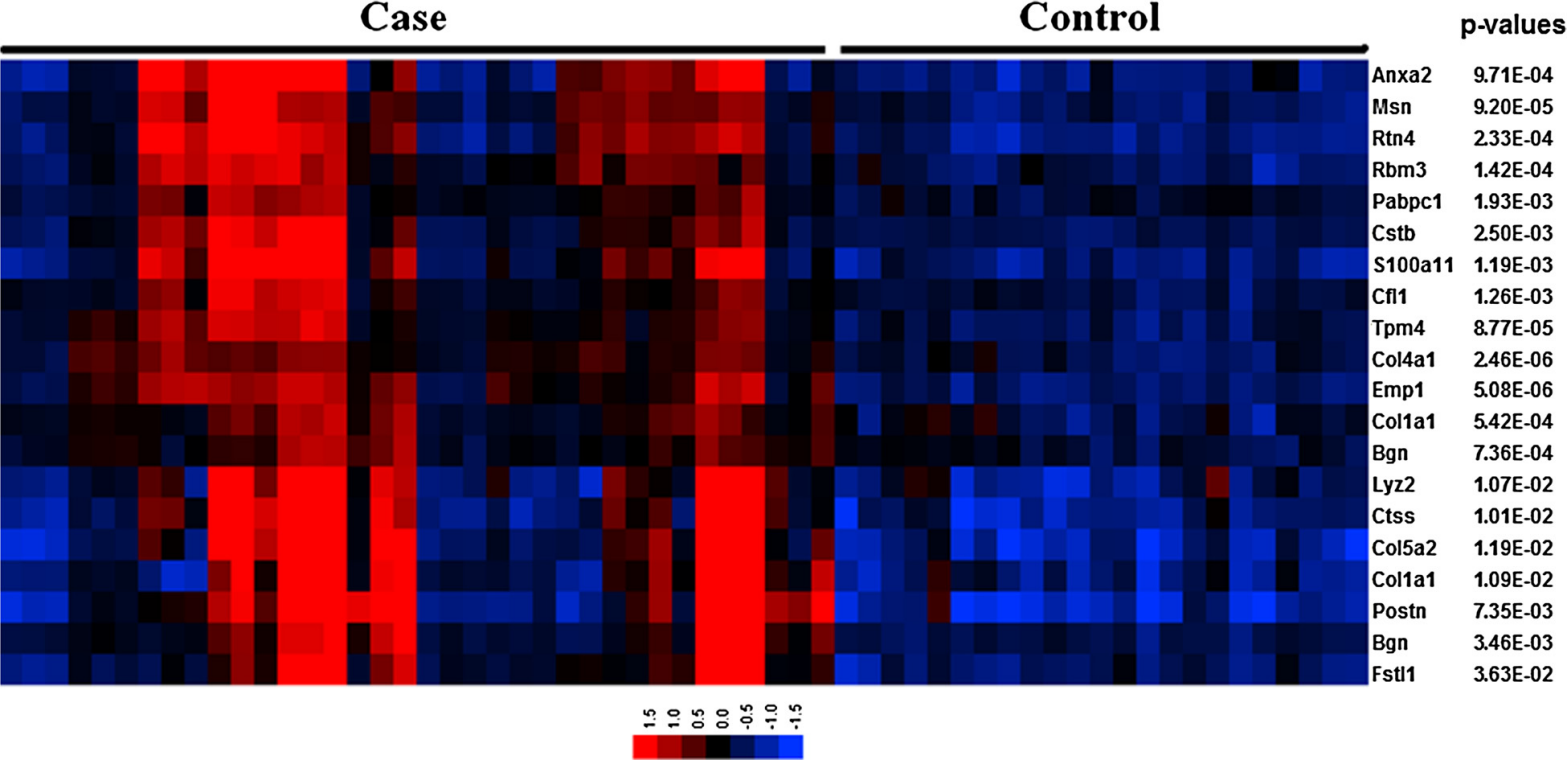
**Gene expression patterns of top candidate community.** Expression values are color-coded: from low (blue) to high (red), and levels of differential expression are shown as adjusted P values.

One of the top candidate communities (*q* = 1E-4) showed a statistically detectable association with extracellular matrix re-organization and angiogenesis, and other processes relevant to cardiac repair after MI. In particular, the Gene Ontology (GO) biological process terms: extracellular matrix organization (P = 0.004), organ morphogenesis (P = 0.01) and blood vessel development (P = 0.02) were highly represented in this community. This community is defined by 18 genes with diverse, but strong co-expression relationships between them (all with ρ > 0.85; Figure 2C). Moreover, the global expression pattern of this community offered indication of its potential disease discriminatory capability (Figure 3). In this signature, those MI samples showing relatively lower expression values (Figure 3) represent those cases derived from mice at earlier times after MI (< 4 hrs). We also note that this community is highly enriched in genes known to be expressed in both the heart (P = 0.007) and blood plasma (P = 0.08) (David tool analysis). All these observations led us to further investigate this top candidate community.

At the center of this community, *Col5a2* displays a relatively large number of connections, which suggests a potential influential role. Prior to this research, *Col5a2* had not been specifically linked to ischemic injury and has not been widely characterized in other domains.

Within this community, other genes are functionally related to *Col5a2*. The following GO annotations are shared by *Col5a2* and the other genes (P < 0.0001): collagen fibril organization (*Anxa2*, *Col1a1*), extracellular matrix structural constituent (*Col1a1*, *Col4a1*), proteinaceous extracellular matrix (*Anxa2*, *Bgn*, *Col1a1*, *Col4a1*, *Postn*). Other collagen genes found to be significantly deregulated are: Col4a1 (adjusted P = 1.3E-07), Col4a2 (P = 4.7E-07) and Col1a1 (P = 7E-05).

The network topological properties of *Col5a2* and the potential novelty of this finding further motivated us to choose this gene as our top prediction. To further assess its potential relevance and to put it in a clinically-related context, we investigated the disease discriminating capacity of this gene in different sample cohorts.

### *Col5a2* accurately distinguishes disease phenotypes

**Figure 4:**
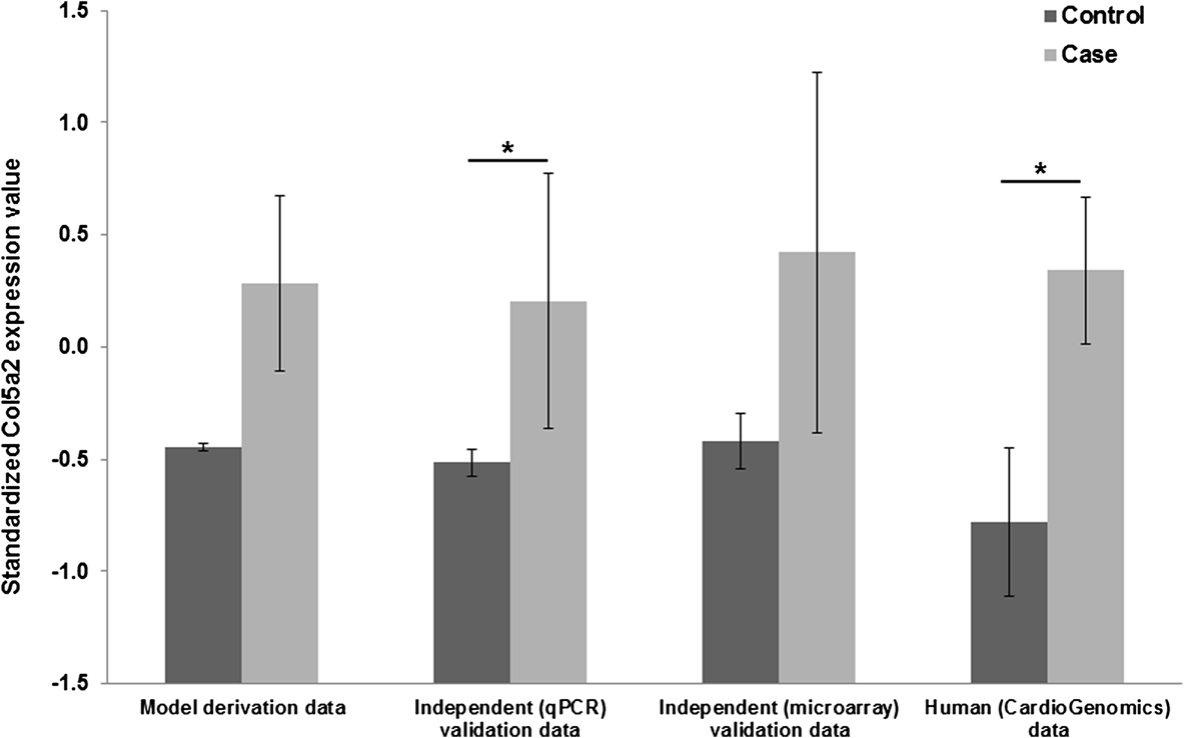
***Col5a2 *expression values in multiple independently generated datasets.** * denotes significant differences between mean values observed in control and case groups at the P = 0.05 level. Vertical bars represent 95% confidence intervals. Case groups represent disease categories.

**Figure 5:**
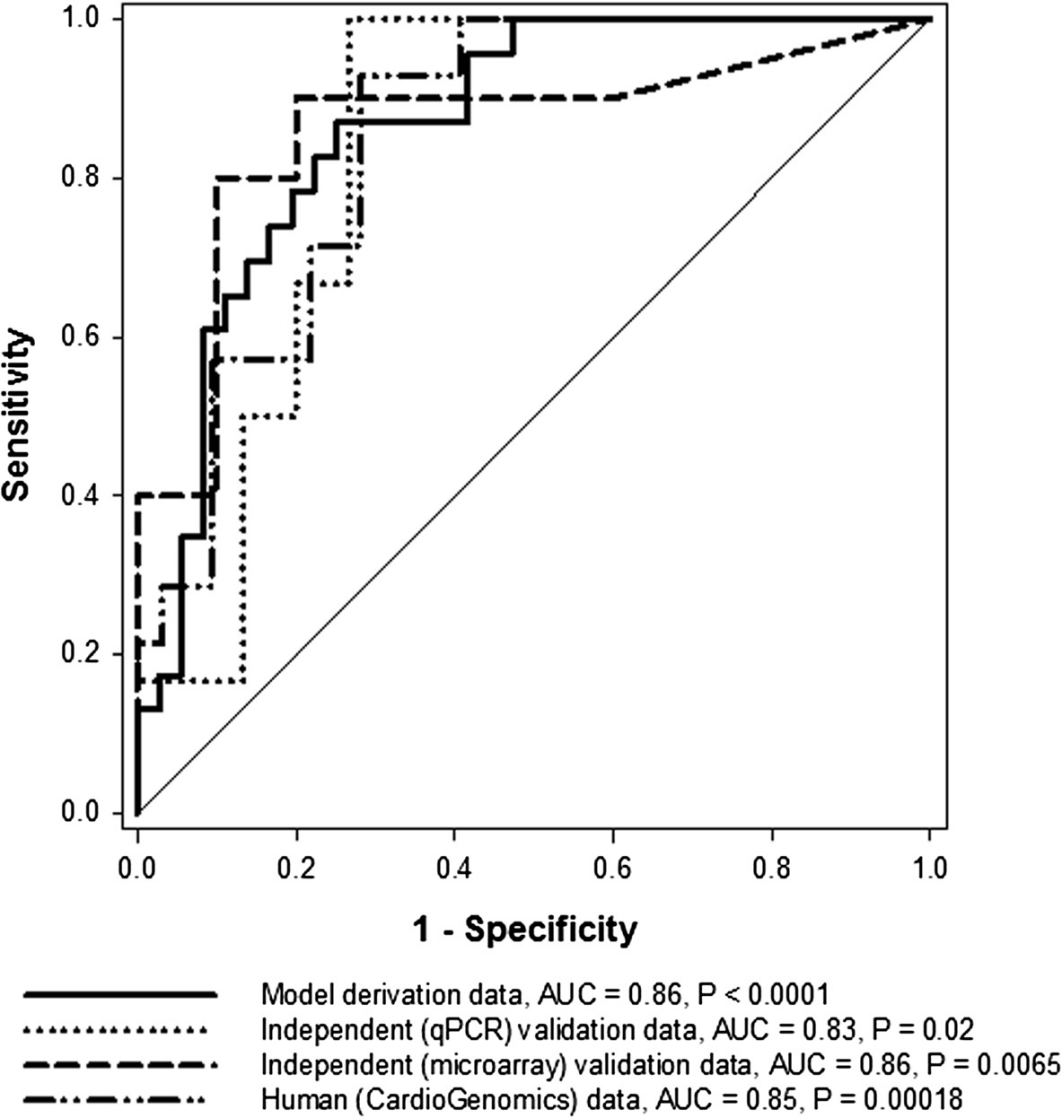
**Disease phenotype discriminatory capacity of a model in which *Col5a2 *expression is used as input.** ROC (receiving operating characteristic) curves obtained when applying the model on independently generated datasets are shown. Diagonal line represents classification performance obtained from a random classification model. P values associated with AUCs estimate their statistical significance in relation to random model. ROC curves and AUC values shown refer to test results on: derivation, independent qPCR, independent mice microarray and human microarray datasets.

*Col5a2* was over-expressed in MI samples in relation to the mean value observed in control samples, though not at the level of P = 0.05 (summarized in Figure 4 as “model derivation data”). Despite this relatively weak differential expression, the disease discriminatory capability of *Col5a2* was demonstrated when using it as an input to a relatively simple classification model (Methods). This model correctly distinguished MI from control samples in the derivation dataset with an AUC = 0.86 (P < 0.0001 vs. random model, Figure 5). This indicated that *Col5a2* expression may accurately reflect pathophysiological effects or events characterizing MI.

### Independent evaluation on qPCR data

To validate the observed *Col5a2* transcriptional responses, we independently measured its expression in myocardial tissue in another mice cohort (Methods). As previously shown in the model derivation dataset, *Col5a2* is similarly over-expressed in the MI samples in relation to the control group (Figure 4, P < 0.05). After standardizing the qPCR data, we next applied the previously obtained classification model to this independent dataset. The classification capacity of the model was remarkably concordant with that obtained on the derivation dataset (tested on qPCR data, AUC = 0.83, P = 0.02 in relation to a random model, Figure 5). This provided additional evidence of both the discriminatory capacity and robustness of *Col5a2* in the MI setting.

### Further independent evaluations on public data

Motivated by our results, we further assessed the potential relevance of *Col5a2* in MI by estimating its disease discriminatory capability in previously generated microarray datasets (Methods). First, we analyzed a (MI vs. control) microarray dataset from myocardial tissue of mice (Methods). As verified before, the expression of *Col5a2* tends to be higher in MI samples (P > 0.05, Figure 4). We applied the classification model obtained above on this independent dataset. This was done after standardizing expression values in the validation dataset, i.e., expression values in the derivation and validation datasets were placed on the same value scales (Methods). The model again showed a substantial capacity to distinguish between MI and control samples (tested on independent mice microarray dataset, AUC = 0.86, P < 0.0065 vs. random model, Figure 5).

To explore the potential pathophysiological role of *Col5A2* in humans, we analyzed publicly available microarray data acquired from cardiac tissue samples of patients with ischemic cardiomyopathy and controls (Methods). Although it does not explicitly compare MI vs. control groups as in our animal models, this high quality dataset offered a good opportunity to estimate the potential clinical application value of *Col5a2*. Again the expression of this gene was elevated in the disease category (Figure 4, P < 0.05), in concordance with our previous results in the MI animal model. More interestingly, when we applied the mouse-derived model on this dataset, after data standardization, a significant and highly concordant classification performance was obtained (tested on human microarray dataset, AUC = 0.85, P = 0.00018 vs. random model, Figure 5).

We also independently tested *Hmox1* (heme oxygenase 1). We chose it as this gene is an example of a statistically differentially expressed gene in our derivation dataset (adjusted P = 0.0008, up-regulated in MI). Also its diagnostic or prognostic value in MI has not been established, though it has been previously linked to atherosclerosis [22]. Moreover, *Hmox1* was a candidate community hub (10 connections). *Hmox1* did not pass our independent validations. Unlike *Col5a2*, the direction of *Hmox1’s* transcriptional response and its classification capacity were not reproduced. In the human dataset, for example, this gene was found down-regulated and offered lower classification capacity.

## Discussion

We showed how a network-based approach can: a. enable the discovery of new biologically meaningful knowledge, and b. provide the basis for potential new clinical applications. At the center of our approach is the detection of highly transcriptionally compact gene communities in a gene co-expression network in MI. The analysis of one such community highlighted the prominent role of *Col5a2*, a gene hitherto not linked to the MI setting. We demonstrated how the disease discriminatory capacity of this gene was both highly accurate and robust across independently generated datasets. After independently validating these findings, we also reported the potential relevance of this classification model in humans. Our research highlights that systems approaches not only can aid in clinically motivated knowledge discovery, but also it offers opportunities for the identification of candidate biomarkers or targets with potential therapeutic benefits. Our findings contribute further evidence of the predictive power and reproducibility of insights resulting from systems-based approaches [23,24].

We focused our attention on Col5a2 because it was included in our top candidate community. Moreover, within this community Col5a2 can be defined as a hub, with 11 strong connections. Lastly, our interest was increased as this gene has not been widely characterized in cardiovascular disease. We did not choose this gene based on its differential expression. If we had followed such a procedure, there would not have been a significant reason to focus on it above the hundreds of differentially expressed genes that can be found in the data.

The extracellular matrix of the myocardium is mainly composed of collagens. These proteins constitute a complex biological interaction network that is key to maintain the structural architecture of the heart and its blood pumping capacity. Following MI, fibroblasts and myofibroblasts enhance collagen synthesis and deposition in the infarcted area in order to strengthen the myocardium and minimize its dilation. Excessive accumulation of collagen in both the infarcted and non-infarcted areas can however lead to ventricular stiffness and heart failure [25]. Several types of collagen have been identified in the heart so far [26-28]. Among them, collagens 1 and 3 are the most widely expressed, representing approximately 90% of the heart collagens. Although collagen 5 represents a small proportion of cardiac collagens (less than 5%), this gene is known to play an important role in the assembly of collagen 1-containing fibrils [29,30]. The collagen 5 molecule has a triple-helix structure that can be defined by different chains: a1, a2 and a3. While expression of col5a1 is detected in the ventricular myocardium, no significant clinically relevant expression of *Col5a2* has been reported in this tissue [28,31]. In our data, collagens 1 and 3 were up-regulated in the MI samples, and their MI-specific expression levels were higher than those of *Col5a2*. However, Col5a2 consistently showed larger (MI vs. control) fold-changes than those observed in collagens 1 and 3.

The link between the expression of *Col5a2* and MI, or related cardiovascular responses, has not been reported to date, though the impairment of collagen 5 expression seems to affect the activity of the main structural collagens of the heart [32]. Using a systems-based approach, here we show for the first time that *Col5a2* expression is critically perturbed in MI. This opens the possibility for using this gene as a new biomarker or therapeutic target of MI and its subsequent pathophysiological responses.

It is noteworthy to stress that *Col5a2* is not highly (statistically) differentially expressed in the derivation cohort at the level of P = 0.05. This underlines the capacity of a system-based approach to generate potential biologically meaningful hypotheses, which go beyond the traditional and often misinterpreted idea of finding genes with “significant” individual differential expression. More important, this corroborates that strong differences in mean expressions are neither necessary nor sufficient conditions to achieve good discriminatory capacity of disease phenotypes. Such an assumption has been traditionally made to study new potential targets and markers in cardiovascular disease.

In the healthy adult myocardium, collagen 1 is mostly expressed around muscle fibers while collagen 5 is mainly detected in the vascular matrix. In the infarcted heart, however, collagen 1 is predominantly expressed in the epicardium and the pericardium that extends into the infarcted myocardium, while collagen 5 is mostly expressed in the peri-infarcted region of the myocardium, surrounding viable myofibers [33]. Collagen 5 may thus play a role in ventricular remodeling following MI, probably by regulating the formation of collagen 1-containing fibers thereby influencing myocardium healing. Nevertheless, the role of *Col5a2* in MI still remains to be fully characterized. Previous research has shown that *Col5a2* seems to be exclusively expressed in the heart valves [28,31,34,35]. Transgenic mice expressing a non-functional form of *Col5a2* do not present ventricular defects [32]. Moreover, patients suffering from classic Ehlers-Danlos syndrome, a rare connective tissue disorder mainly caused by mutations in *COL5A1* or *COL5A2*, do not appear to show ventricular malformations [36]. However, mutations in *Col5A2* have been associated with vascular disease, such as cervical artery dissection [37] and aortic dissection [38].

Our investigation showed that *Col5a2* is highly expressed in the left ventricle after MI. This indicates that at least one of the different collagen 5 isoforms containing the a2 chain may be required during post-MI response, most probably to allow synthesis and deposition of sufficient amounts of collagen 1 in the infarcted area. Despite the potential relevance of this finding, additional research will be needed to define the specific role of *Col5a2* in heart repair after MI, as well as its potential diagnostic or prognostic value.

It has recently been observed that *Col5a2* is highly expressed in invading neoplastic epithelial cells [39], and that it is expressed in the human fetal gut and in colon cancer cells [40]. This confirms that *Col5a2* is linked to higher extracellular matrix turnover. Furthermore, *Col5A2* has been associated with lymph node metastasis in lung adenorcarcinoma [41]. Experiments in tendon cells [42] and fibroblasts [43] have shown that *Col5a2* plays an important role in guiding cell proliferation.

A potential limitation of our investigation is that the model derivation dataset included samples obtained at different time points ranging from 1 hour to 8 weeks after MI [10]. This constrains the potential implications of our findings in the context of MI diagnosis and post-MI prognosis. Nevertheless, we were able to demonstrate both the predictive accuracy and robustness of *Col5a2* in different independent datasets and experimental platforms. This underscores the possible relevance of our results to the ischemic heart disease context in general. Another aspect that deserves further investigations is the integrated analysis of the *Col5a2*-centric community identified by our approach (Figure 3). Limitations to experimentally measure all the genes involved this community prevented us from validating their integrated predictive capability here. We note, however, that our computational analysis also indicates the disease discriminatory capability of this community in the derivation dataset (Figure 3). Another potential limitation is that candidate biomarkers obtained from tissue samples may not necessarily translate into useful circulating plasma biomarkers. Lastly, future investigations will require comparisons with standard biomarkers, such as troponin levels. In this article we did not report additional comparisons due to lack of access to these measurements in the published studies and due to limited amounts of our samples.

## Conclusions

Our systems-driven approach revealed a novel critical predictive role of *Col5a2* in MI. This brings *Col5a2* to the pipeline of candidate biomarkers and targets with potential therapeutic benefit. Our network-based discovery strategy may have broad applications for studying other disease phenotypes. Based on this approach we probed a novel association between *Col5a2* and its community of tightly co-expressed genes with MI. In the long term, *Col5a2* may represent a new prognostic or therapeutic target for patients suffering ischemic heart disease. Additional independent analysis, including those involving tissue-derived and circulating proteins, will be required to further elucidate functional and predictive roles of *Col5a2*.

## Competing interests

The authors declare that they have no competing interests.

## Authors’ contributions

FA conceived the study, developed the A-CODE algorithm, contributed computational analyses and drafted the manuscript with the support of all the co-authors. LZ contributed data analyses, including classification models. CJ performed qPCR experiments and assisted with their analysis. SLP implemented MI model in mice and contributed samples for independent validation. SR supported experimental validation and provided biological insights. DW contributed to evaluation of findings, including clinical interpretations. All authors read and approved the final manuscript.

## Pre-publication history

The pre-publication history for this paper can be accessed here:

http://www.biomedcentral.com/1755-8794/6/13/prepub

## Supplementary Material

Additional file 1 Supplementary methods. Click here for file

Additional file 2 **Mouse validation (qPCR) data.** Quality and quantification of *Col5a2* RNA. Click here for file

Additional file 3 Minimum information of qPCR experiments based on MIQE guidelines. Click here for file

Additional file 4 **Co-expression network in MI.** First two columns represent interacting genes, third column shows co-expression values. Click here for file

Additional file 5 Examples of top-ranked candidate communities. Click here for file
