# Supplementary material for: Analysis of a gene co-expression network establishes robust association between Col5a2 and ischemic heart disease
Source: BMC Med Genomics. 2013 Apr 10;6:13. doi: 10.1186/1755-8794-6-13 (PMC3637268; doi:10.1186/1755-8794-6-13)
Supplement: Additional file 1 — Supplementary methods. [file 1755-8794-6-13-S1.pdf]

Analysis of a Gene Co-expression Network Unveils Influential Role of Collagen 5a2 in ischemic heart disease

Francisco Azuaje[1], Lu Zhang[2], Céline Jeanty[2], Sarah-Lena Puhl[3], Sophie Rodius[2], Daniel Wagner [2,4]

[1] NorLux Neuro-Oncology Laboratory, Department of Oncology, CRP-Santé, Luxembourg, Luxembourg.
[2] Department of Translational Cardiovascular Research, CRP-Santé, Luxembourg, Luxembourg.
[3] Department of Internal Medicine III, Saarland University Hospital, Homburg, Germany.
[4] Division of Cardiology, Centre Hospitalier, Luxembourg, Luxembourg.

Supplementary Methods

Animal model

Male C57/Bl6N mice, aged 12-14 weeks, were kept under usual care, fed ad libitum on standard laboratory mice chow and had free access to water. Myocardial infarction was induced by permanent ligation of the left anterior descending coronary artery (LAD). Mice were anesthetized with a 1:10 dilution (diluted with 0,9% NaCl) of a mixture of Ketaminhydrochlorid (100 mg/kg) and Xylazinhydrochlorid (10 mg/kg). Ten minutes after administration, movement of whiskers and reflexes was tested. Lack of reaction ensured a stable and deep sedation for about 40 minutes. Mice were euthanized by an intraperitoneal application of an undiluted mixture of Ketaminhydrochlorid (100 mg/kg) and Xylazinhydrochlorid (10 mg/kg).

Animals were placed in a supine position on a heating pad (37°C) intubated with a 22-gauge stump needle and ventilated with room air using a MiniVent mouse ventilator (Hugo Sachs Elektronik, Germany; stroke volume 200 µl, respiratory rate 125 strokes per minute). The LAD was ligated approximately 2 mm below the left atrium using a 6-0 silk suture.

Occlusion of the LAD was confirmed microscopically by discoloration of the ischemic area below the ligation-node. Sham-operated animals underwent the same procedure without occlusion of the LAD. After the surgical intervention, mice were kept again under usual care for 4 weeks.

Determination of collagen content

For histological analysis, transverse mid-ventricular tissue slices were fixed with 4% formaldehyde and paraffin-embedded. Five µm paraffin slices were stained with Sirius Red to detect connective tissue. LUCIA software (Nikon) was used to determine degree of fibrosis in the infarction area. The animals were categorized in groups with a collagen content of 15-40% (small infarction), 41-55% (mid-sized infarction) and 56-85% (big infarction) in the anterolateral wall.

RNA samples

Total RNA was extracted from frozen tissue samples with a TRIzol (Invitrogen, Carlsbad, CA) isolation protocol. Homogenisation of samples was performed with a Polytron® (Bohemia,USA) and insoluble material from the homogenate was removed by centrifugation at 800rcf for 10min at 4°C. Total RNA was purified with a RNeasy mini kit combined with an on-column DNase treatment following the manufacturer's instructions (Qiagen, Valencia, CA). RNA quantity was assessed with a Nanodrop (Thermo Scientific, Wilmington, USA) and quality was evaluated using the Agilent 2100 Bioanalyzer (Agilent Technologies, Palo Alto, CA). RNAs used in the present study were of good quality and un-degraded. See

Supplementary File 2 for quality RNA samples. All nucleic acid samples were stored at -80°C until use.

Reverse transcription

1µg of RNA were reverse transcribed into cDNA using the SuperScript II reverse transcriptase with the following protocol: RNAs were mixed with the 5X RT buffer, random hexamers, dNTPs and DTT in a total volume of 19µl. Samples were then heated to 42°C for 2 min, and 1µL of SuperScript II was added to a total volume of 20µl. RT was allowed for 50 min at 42°C and was followed by enzyme inactivation at 70°C for 15 min. Final concentrations were: 50mM Tris-HCl, 75mM KCl, 3mM MgCl2, 180ng of random hexamers, 0.5mM dNTPs, 10mM DTT, 200U of SuperScript II (Invitrogen). In each RT-PCR a no template control (no RNA in RT) was implemented.

Quantitative real-time PCR (qPCR)

cDNAs obtained from RT of RNA were diluted 10-fold and 4µL were mixed with 16µL of SYBR®Green Master Mix (Biorad, Nazareth, Belgium) containing 300nM of each primer (final volume 20µL). Amplification was carried out in the CFX96 thermal cycler (BioRad) under the following conditions: heating for 3 minutes at 95°C, 40 cycles of denaturation for 30 seconds at 95°C, followed by an annealing/extension for 1 min. After each run a Melting curve analysis was performed, ramping from 55°C to 95°C in 20min. A negative control without cDNA template was run in every assay and measures were performed in duplicates.

Intron-flanking primers were designed with the Beacon Designer Pro 7.8 software (Premier Biosoft, Palo Alto,USA). Specificity was assessed using the NCBI BLAST tool [1]. Melting curves were analyzed and amplicons were observed on agarose gel to confirm the specificity of the reaction.

Expression levels were calculated using the CFX manager 2.1 software (Biorad) via the delta-Cq method, incorporating the calculated amplification efficiency for each primers pair. See Supplementary File 2 for Raw Cq data and Supplementary File 3 for MIQE checklist.[2]

A-CODE algorithm for network community detection

This approach is based on the notion that strong communities are built around strong edges in the community. Moreover, candidate communities should also represent tightly interconnected webs of neighboring relationships (Figure S1). Thus, A-CODE searches for strong, highly-interconnected communities around each edge in the network (Figure S2).

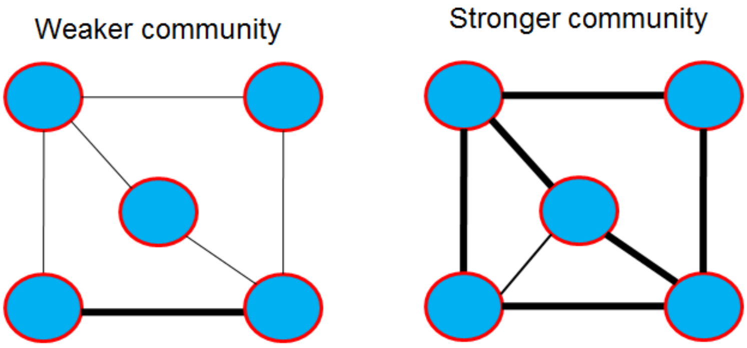

Figure S1. Illustration of the concept of strong candidate community in weighted networks with A-CODE. Strong communities are based on strong and highly connected relationships among their members. The thickness of the lines reflects the relationships strength, e.g., co-expression value.

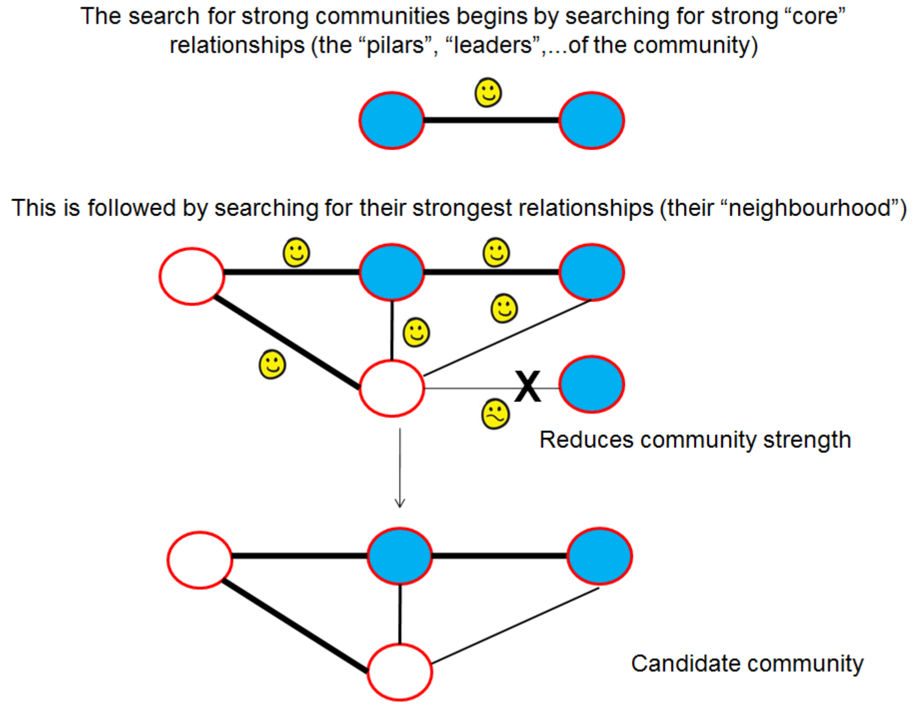

Figure S2. Illustration of the computational notion of network community detection with A-CODE.

Candidate communities are characterized by their co-expression *compactness*, which is here based on the mean co-expression value observed in the candidate community. To reduce possible bias towards highly variable co-expression patterns, compactness is computed as the mean co-expression value divided by the standard deviation of the values found in a candidate community. The expected rate of false discoveries, q, for each observed compactness value is computed with a statistical test based on random permutations. Thus, strong candidate communities are those displaying high co-expression compactness with corresponding low q values. At each search step, A-CODE adds a new edge to the candidate community. Each new edge is derived from the direct neighborhood of the current candidate community. Similarly, at each search step the neighboring edge with the highest co-expression value, ρ, is selected for inclusion. This process continues until either a minimum q

(min_q) cannot be obtained or until a maximum number of edges in the candidate community has been reached. Experiments reported here are based on min_q = 1E-4 and a maximum number of 20 edges in each candidate community. The latter was suitable to assist expert visualization and interpretation. Also the min_q value selected is stringent enough to filter out communities for which more than 1 permutation experiment (out of 10000 implemented) reported compactness values equal or higher than that observed in the candidate community. At the end of this process, each network edge gives rise to a candidate community.

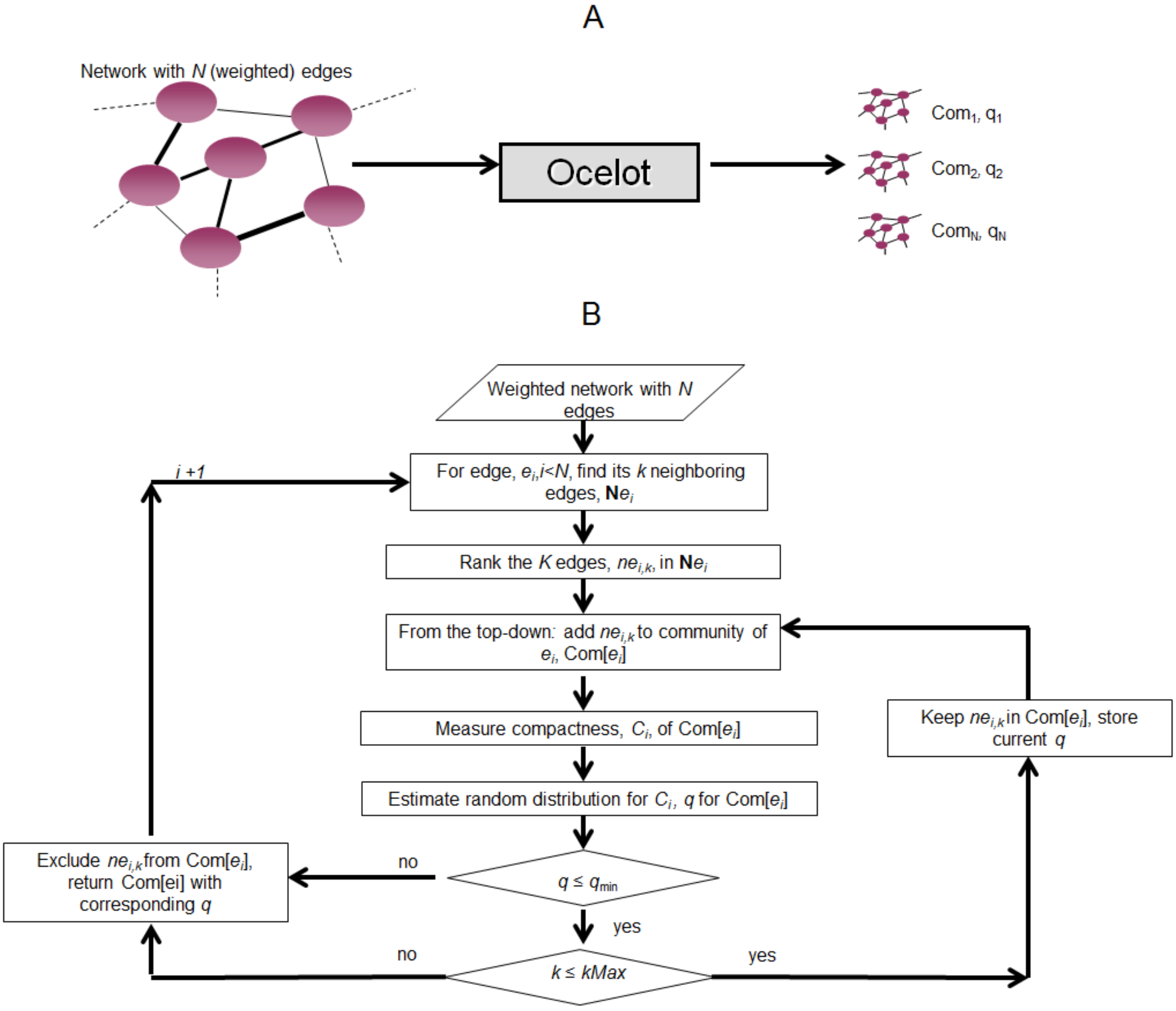

Figure S3. The A-CODE algorithm. A. Illustration of input/output scheme. B. Flowchart of community detection procedure.

References

1. BLAST: Basic Local Alignment Search Tool. 2012; Available from: http://www.ncbi.nlm.nih.gov/BLAST/Blast.cgi

2. Bustin SA, Benes V, Garson JA, Hellemans J, Huggett J, Kubista M, et al.The MIQE guidelines: minimum information for publication of quantitative real-time PCR experiments. *Clin Chem* 2009;**55**:611-622.
