# Supplementary material for: Analysis of a gene co-expression network establishes robust association between Col5a2 and ischemic heart disease
Source: BMC Med Genomics. 2013 Apr 10;6:13. doi: 10.1186/1755-8794-6-13 (PMC3637268; doi:10.1186/1755-8794-6-13)
Supplement: Additional file 2 — Mouse validation (qPCR) data. Quality and quantification of Col5a2 RNA. [file 1755-8794-6-13-S2.pdf]

Quality and quantification of Col5a2 RNA

Intervention	Nanodrop (A260/A230)	Nanodrop (A260/A280)	Bioanalyzer (RIN)	Mean Raw Cq value (GAPDH)	Mean Raw Cq value (Col5a2)	Pre-processed data
Sham	2.25	2.09	6.9	14.65	23.96	1.58E-03
Sham	1.03	2.09		14.57	24.38	1.11E-03
Sham	2.17	2.07	7.5	14.76	24.4	1.25E-03
Sham	2.25	2.09	7	14.47	24.6	8.33E-04
Sham	1.59	2.09	7.9	14.54	24.69	8.80E-04
Sham	1.42	2.1	8.4	14.56	24.86	7.93E-04
Infarction	2.08	2.09	7.1	15.55	21.53	1.58E-02
Infarction	2.22	2.04	7	15.69	22.01	1.25E-02
Infarction	2.22	2.05	7.3	15.01	22.64	5.05E-03
Infarction	1.71	2.06		14.57	22.68	3.62E-03
Infarction	2.29	2.08	7.9	15.03	22.96	4.10E-03
Infarction	2.1	2.09	7.5	14.57	23.06	2.78E-03
Infarction	2.14	2.06		14.5	23.27	2.29E-03
Infarction	2.26	2.07	7.9	14.35	23.6	1.64E-03
Infarction	2.26	2.1	7.8	14.52	23.76	1.65E-03
Infarction	2.18	2.06	6.4	16.36	24.52	3.50E-03
Infarction	2.22	2.08	7.8	14.92	24.57	1.24E-03
Infarction	1.46	2.05		14.37	24.6	8.92E-04
Infarction	2.29	2.09	7.8	14.6	24.68	9.24E-04
Infarction	0.92	2.09	7.6	14.71	24.92	8.44E-04
Infarction	1.64	1.82	7.1	16.34	25.5	1.75E-03
